# Development of a Real-Time Controlled Bio-Liquor Circulation System for Swine Farms: A Lab-Scale Study

**DOI:** 10.3390/ani11020311

**Published:** 2021-01-26

**Authors:** Seungsoo Kim, Arif Reza, Soomin Shim, Seunggun Won, Changsix Ra

**Affiliations:** 1 Department of Animal Industry Convergence, College of Animal Life Sciences, Kangwon National University, Chuncheon 24341, Korea; seungsoo89@kangwon.ac.kr (S.K.); reza.arif@kangwon.ac.kr (A.R.); smshim@kangwon.ac.kr (S.S.); 2 Department of Animal Resources, College of Life and Environmental Science, Daegu University, Gyeongsan 38453, Korea; swon@daegu.ac.kr

**Keywords:** bio-liquor circulation, real-time control, oxidation-reduction potential, pH, odor, NH_3_, swine manure

## Abstract

**Simple Summary:**

Odor emission from swine production facilities can irritate the people living in surrounding areas, although the farmers consider odor emission as a part of farming practice. Despite the governmental and institutional efforts, odor-related complaints from the neighborhood communities around the swine farms are rapidly increasing and have been identified as a key concern to sustaining progress of the swine industry globally. Bio-liquor circulation systems (BCSs) in swine farms have become popular among the farmers as an odor reduction technology in Korea. However, due to the lack of appropriate operating strategies, the odor reduction capacity of BCSs is often depleted. In this lab-scale study, a real-time control strategy based on oxidation–reduction potential (ORP) and pH (mV) time profiles was developed and applied for BCS operation. This study shows the potential effectiveness of using ORP and pH (mV) time profiles as operational parameters for the BCS to improve swine manure properties in slurry pits and thus reduce odor emission.

**Abstract:**

In this study, an attempt was made to develop a real-time control strategy using oxidation–reduction potential (ORP) and pH (mV) time profiles for the efficient operation of bio-liquor circulation system (BCS) in swine farms and its effectiveness in reducing odor emission through improving manure properties in the slurry pit was evaluated. The lab-scale BCS used in this study comprised a bioreactor and a slurry pit. The bioreactor was operated in a sequence of inflow of swine manure → anoxic phase → aerobic phase → circulation to the slurry pit. The improvement in swine manure properties was elucidated by comparing the results of the BCS slurry pit (circulation type, CT) and conventional slurry pit (non-circulation type, NCT). The results revealed that the ORP time profile successfully detected the nitrate knee point (NKP) in the anoxic phase. However, it was less stable in detecting the nitrogen break point (NBP) in the aerobic phase. The pH (mV) time profile showed a more efficient detection of NBP. Compared to the NCT slurry pit, concentrations of ammonium nitrogen (NH_4_-N) and soluble total organic carbon (STOC) and other analyzed swine manure properties were much lower in the CT slurry pit. In the aspect of odor reduction, around 98.3% of NH_3_ was removed in the CT slurry pit. The real-time controlled BCS can overcome the drawbacks of fixed time-based BCS operation and therefore can be considered as a useful tool to reduce odor emission from intensive swine farming operations. However, further studies and refinement in control algorithms might be required prior to its large-scale application.

## 1. Introduction

Intensive livestock farming systems inevitably result in numerous environmental concerns in many parts of the world [1]. Complaints regarding odor emission from livestock farms have been increased significantly worldwide and become the prime concern for livestock industries [2,3]. The problems related to odor emission from livestock facilities in Korea are visible through the trend of press releases in internet news stories. In 2010, there were around 462 articles related to odor emission from livestock farms, whereas it reached 5566 in 2018 [4]. In addition, according to the Ministry of Environment (MoE) and the Ministry of Agriculture, Food and Rural Affairs (MAFRA), 3061 (about 46%) out of 6712 cases of odor complaints in Korea between November 2009 and October 2013 were related with livestock farms [5]. Another report mentioned that 2838 out of 10,753 odor complaints were associated with the livestock industries in 2015, which was the highest among all the 18 surveyed sectors, and the majority (46.0%) of the odor-related complaints were associated with swine farms [6]. Moreover, among the various complaints, including odor, noise, unauthorized discharge of waste filed to Anti-Corruption and Civil Rights Commission (ACRC), Korea in 2017, chronic stress caused by odor emitted from swine farms was identified as one of the main issues [7]. Therefore, social conflicts related to odor emission are expected to intensify gradually and urgent countermeasures are required to reduce odor emission from swine farms. 

Various odor removal facilities have been developed [3,8,9], but more than 168 odor-inducing substances can be generated from swine manure [10]. These odorous substances belong to four chemical groups: (1) volatiles fatty acids; (2) aromatic compounds; (3) nitrogen compounds; and (4) sulfur compounds [11,12]. In general, nonionic substances having a molecular weight of less than 300 are easily volatilized and cause odor [13]. Decomposition of high molecular organics to low molecular organic compounds by the microbial action is considered as the main cause of odor from swine manure. The odor from swine farms originates from the incomplete anaerobic decomposition of nutrients in undigested manure, endogenous secretions of animals, and intermediate and end products of intestinal microorganisms stored for a long time [11,14,15,16,17]. Therefore, it is not easy to minimize odor from swine farming practices. Although well-known odor removal technologies such as absorption towers, bio-filters, bio-scrubbers, and bio-curtains can remove some odor from the swine barn, none of the aforementioned facilities are targeted towards controlling the odor at the generation sources; odor source control is one the basic principles of odor reduction and considered as the first priority [11]. Ammonia (NH_3_) is one of the odorous gases emitted from the swine farming system and affects the respiratory tract of farmworkers as well as the growth of animals [18]. In the available literature regarding NH_3_ as an odor indicator, some mixed results were found. Some studies indicated NH_3_ as a poor indicator of odor emission [19,20,21,22], whereas contemporary studies reported the inhibition of NH_3_ emission with simultaneous odor reduction [23,24,25]. In general, NH_3_ concentration in swine barn is proportional to the amount of protein and amino acids decomposed, and so is the odor emission [26,27]. Therefore, the minimization of NH_3_ concentration along with manure properties improvement in swine farms might indicate a reduction in odor emission.

The conventional recirculation systems for manure use either water or treated manure, where the entire shallow slurry pit is drained (batch mode) and recharged with anaerobically treatment lagoon effluent [28,29,30,31]. However, in some parts of the world, water is a precious resource and, thus, can be problematic when used for flushing in swine farms—it increases manure volume [31]. Moreover, treating manure in lagoons also requires a large amount space, which is not feasible for countries with limited land resources such as Korea. This recirculation system also needs active monitoring and operational support, and hence is not applicable for automated production systems. 

The Korean swine manure management practice is quite different from other parts of the world due to its climate condition and limited land resources [32]. Swine manure in Korea must be demarcated to solid and liquid fraction and then composted before application to arable lands, or purified prior to discharge into the environment. Most of the Korean swine farms possess on-farm manure treatment facilities, and more than 50% of the farms use aerobic biological treatment process for liquid composting [33]. In general, the treatment capacity of the cylindrical shaped liquid composters are above 200 tons. Bio-liquor circulation systems (BCSs) in swine farms are gaining popularity in Korea, due to the odor reduction capability [34,35,36,37], and the fact that the system is easy to connect to the existing on-farm liquid composting or purification process. The term “bio-liquor” refers to biologically treated mixed liquor. Generally, in wastewater treatment process, solids containing microorganisms are called bio-solids and liquors containing microorganisms and some solids are known as mixed liquors. Therefore, it would be reasonable to denote the supernatant of biologically treated swine wastewater which contains enzymes secreted by microorganisms as well as suspended solids as bio-liquor. BCS is a method that improves the swine manure properties in the slurry pit by treating the manure in a bioreactor outside of the swine barn and then recirculating it to the slurry pit and, therefore, is considered as an effective way to control the odor source. However, the system has some drawbacks, including proper system design and established operating conditions for proper system operation. Currently, on-site BCS operating practices are mostly employed using quantitative operation methods based on a predetermined time or circulation rate [37]. Therefore, issues related to low or high loading rate can occur depending on the designed system scale and operating conditions. If such imbalance between the treatment capacity and the loading rate in the bioreactor is sustained for a long time, the swine manure treatment efficiency of the bioreactor could be decreased. Especially, if the high loading rate is maintained for a long time, the bioreactor treatment efficiency will be reduced to zero. Moreover, other common problems such as the (1) inflow of raw manure without separation of solid and liquid fractions; and (2) loss of manure treatment performance of the bioreactor due to improper circulation have been reported by the Korea Pork Producers Association (KPPA) [38]. Hence, standardized process design and proper operating conditions are required for effective BCS operation. However, process optimization is not likely to be easy because the efficiency of the biochemical swine manure treatment process is affected by various factors, including characteristics of manure, temperature, pH condition, microbial activity in the bioreactor, season, and others [39]. Considering the various problems arising from the swine manure treatment process, developing a real-time control system that can track the changes of manure properties in the bioreactor and control the system based on diagnosed results could be an effective solution.

Many new sensor-based and internet of things (IoT) technologies have been developed, and efforts have been made to optimize feeding, breeding, and environmental management by introducing information and communications technology (ICT) to livestock industries. Previous studies have successfully demonstrated the application of real-time control strategy using moving slope changes of oxidation–reduction potential (ORP) and pH (mV) time profiles in treating swine wastewater [40,41,42,43]. Therefore, this study was conducted to develop a real-time control technology based on ORP and pH (mV) time profiles for efficient BCS operation, thus improving swine manure properties in the slurry pit and reducing odor emission.

## 2. Materials and Methods 

### 2.1. Lab-Scale Experimental Set Up

**Figure 1:**
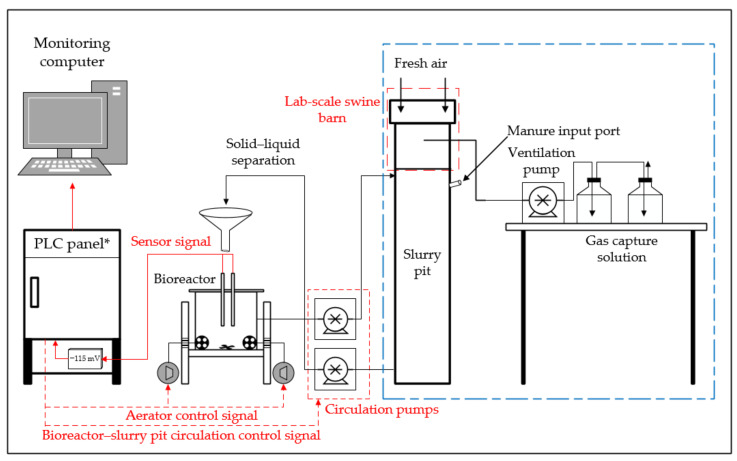
Schematic of the experimental setup. (*PLC, Program Logic Controller)

The schematic of the lab-scale experiment setup is shown in Figure 1. The real-time controlled lab-scale BCS process consisted of a bioreactor and a swine barn with a circulation type (CT) slurry pit (as shown in the black box in Figure 1). The efficacy of the BCS to improve the swine manure properties in the CT slurry pit was evaluated by comparing with a typical slurry pit (non-circulation type, NCT) found in conventional swine farming systems in Korea (as shown in the blue box in Figure 1). In both types, a certain amount of manure was injected once a day into each slurry pit to imitate the swine manure excretion situation of the swine farms.

#### 2.1.1. Bioreactor Construction 

**Figure 2:**
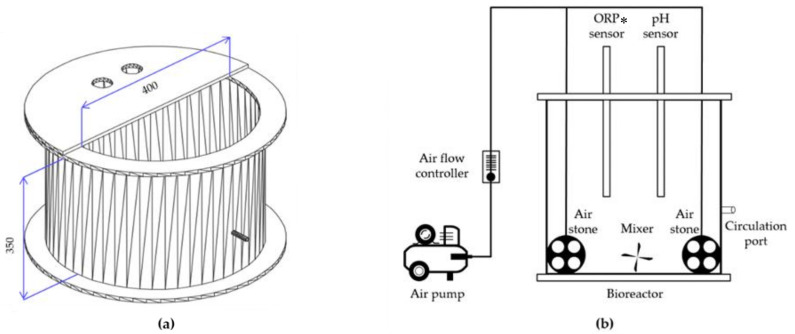
Schematic of the (**a**) bioreactor and (**b**) bioreactor components. (*ORP, oxidation-reduction potential)

The cylindrical-shaped open system bioreactor used in this study was made using acrylic of 10 mm in thickness (Figure 2). The working volume of the bioreactor was 30 L, with a diameter and height of 400 mm and 350 mm, respectively. Two sensor holders were placed on the top of the bioreactor and the ORP and pH sensors were inserted into the reactor through the probe hole of the holder. For efficient process operation, the sensors were cleaned twice in a week and the cleaning was performed after manure circulation. The bioreactor also contained two air stones, and the aeration rate was maintained as 0.05 L/L·min using air flow meters. A magnetic bar was placed at the bottom of the reactor for mixing. For complete mixing, a mixing speed of 200 rpm was used. Furthermore, a circulation port was located at the middle of the bioreactor and connected to the lab-scale slurry pit using a silicon tube.

A sieve cradle was placed on the top of the bioreactor and the solid–liquid separation process was carried out using a sieve (pore size 0.435 mm). The solid–liquid separation sieve was cleaned every 24 h to maintain separation efficiency.

#### 2.1.2. Development of the Monitoring System 

The time-profiles of ORP (sensor number-HI3230B, HANNA instruments, Seoul, Korea) and pH (sensor number-HI3230B, HANNA instruments, Seoul, Korea) were monitored through a program logic controller (PLC, model name-XGB, LS ELECTRIC Co, Gyeonggi-do, Korea). The measuring instrument (ORP, ENVA K401; pH, ENVA K301, KOREA ENVA TECH, Gyeonggi-do, Korea) received the signals of ORP and pH (mV) from the sensors. The measured signals were then relayed as 0~24 mA to the program logic controller (PLC) every second. The signals received in PLC were transmitted to a computer and visualized by the human–machine interface (HMI, AUTOEYE). The bioreactor operation and circulation with the slurry pit were controlled by the PLC connected to the main computer. The PLC traced the ORP and pH (mV) time profiles and operated the whole process using the designated algorithm.

##### Operational Algorithm for Bioreactor 

The bioreactor operation algorithm was designed based on a previous study [39] and referred to some other studies [42,43]. The main steps of bioreactor operation were the inflow of swine manure → anoxic phase → aerobic phase → circulation. After feeding the bio-liquor into the bioreactor and ensuring complete mixing, the computerized program started reading ORP and pH values every second. The calculated average value of 60 data points was recorded every minute for further signal processing. The moving slope change (MSC) of ORP (MSC_ORP_) and pH (mV) (MSC_pH_) time-profiles were set to measure sample sizes of 10 points (*r* = 10) every one-minute and stored in the computer. Details of the MSC process related to bioreactor control using ORP or pH (mV) time profiles can be obtained from previous studies [39,42,43,44]. 

**Figure 3:**
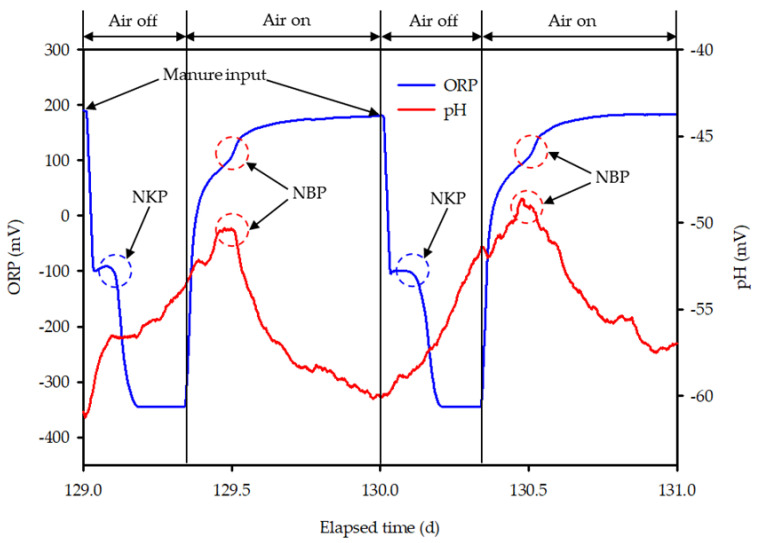
Representative time-profiles of ORP and pH (mV) according to bioreactor operation [39].

For optimization of the bioreactor operation, the denitrification termination point was traced using ORP time-profile in the anoxic phase (Figure 3). In the anoxic phase, the first drop in ORP (FDO) was observed with the inflow of swine manure, and thereafter remained constant during the denitrification process (APP, appearance of plateau point). The second drop in ORP (SDO) indicated the denitrification termination point (NKP, nitrate knee point). The applicability of ORP in recognizing the NKP, four different combinations of FDO, APP and SDO trigger values such as (<−30, >−10, and <−25), (<−30, >−10, and <−15), (<−30, >−5, and <−10) and (<−30, >−10 and <−10), respectively, were used. The PLC sequentially detected the FDO, APP, and SDO, and turned on the aerator when the SDO was detected. However, if the PLC failed to recognize the trigger values, the anoxic phase would last forever. The absolute value of ORP was therefore used as a co-factor to prevent this problem. The ORP value was remarkably decreased below −300 mV after the denitrification reaction was terminated [39]. Therefore, the ORP value as a lower limit was set at −240 mV and the aerator was immediately turned on when the ORP value reached to lower limit, regardless of the MSC_ORP_ in the anoxic phase (MSC_ORP_an_).

**Figure 4:**
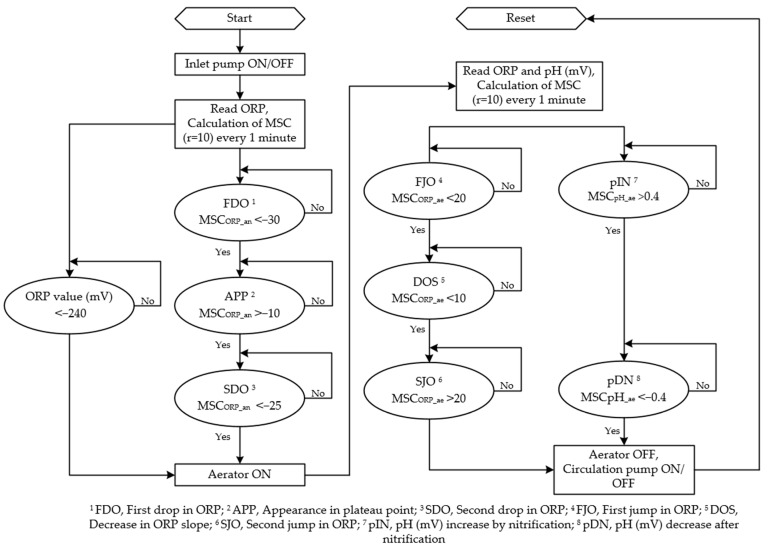
Representative real-time control algorithm of the operational process.

In the aerobic phase, the nitrification termination point (NBP, nitrogen break point) was diagnosed based on the time-profiles of ORP and pH (mV). After starting aeration, the ORP value was sharply increased (FJO, first jump in ORP) and the ORP slope was gradually decreased (DOS, decrease in ORP slope). Then, the second jump in ORP (SJO) was observed when the nitrification was completed. While an increase in pH (mV) was observed with the onset of aeration (pIN, pH (mV) increase by nitrification) and after completion of the nitrification process, the pH suddenly began to decrease (pDN, pH (mV) decrease after nitrification). Two sets of trigger values for FJO, DOS, and SJO, such as (>20, <10 and >20) and (>15, <10 and >20), respectively, were used to recognize the MSC of ORP in the aerobic phase (MSC_ORP_ae_), while another two amalgamations of pIN and pDN trigger values such as (>0.4 and <−0.6) and (>0.4 and <−0.4), respectively, were applied to identify the MSC of pH (mV) in aerobic phase (MSC_pH_ae_). Based on the aforementioned characteristics of the ORP and pH (mV) time profiles, the autonomous bioreactor control algorithm used in this study was designed (Figure 4). After the end of swine manure circulation, the control algorithm needed to be reset and the same process was repeated continuously.

#### 2.1.3. Construction of the Lab-Scale Swine Barn 

**Figure 5:**
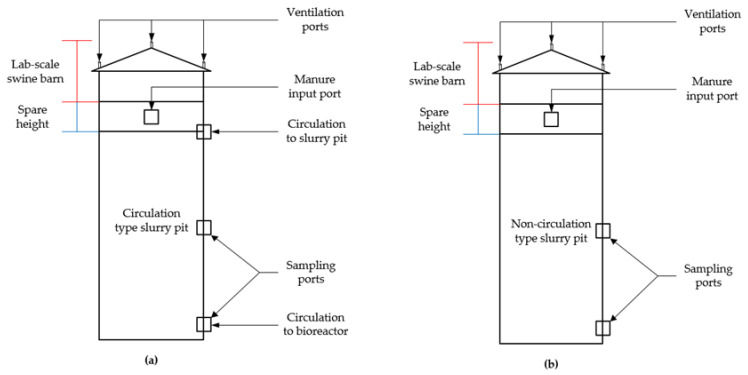
Outline of the lab-scale (**a**) bio-liquor circulation system (BCS) swine barn and (**b**) non-circulation type (NCT) swine barn.

The outline of the lab-scale swine barns is shown in Figure 5. The swine barns were constructed using 10 mm acrylic fiber and designed based on the concept of windowless swine farming systems. In the case of windowless swine farms, continuous ventilation is performed to prevent the accumulation of harmful gases inside the barn. The amount of ventilation air in the lab-scale swine barn was calculated as 4.4, 0.7 and 15.3 m^3^/d for spring and fall, winter, and summer seasons, respectively, using the standard construction guidelines for swine farms provided by KPPA [45]. The detail calculation of the ventilation rate is presented in Table S1. Ventilation ports were installed in the simulated barn, and ventilation was performed using silicone tubing and a peristaltic pump (WT600-3J, Longer Precision Pump Co., Ltd, China).

**Figure 6:**
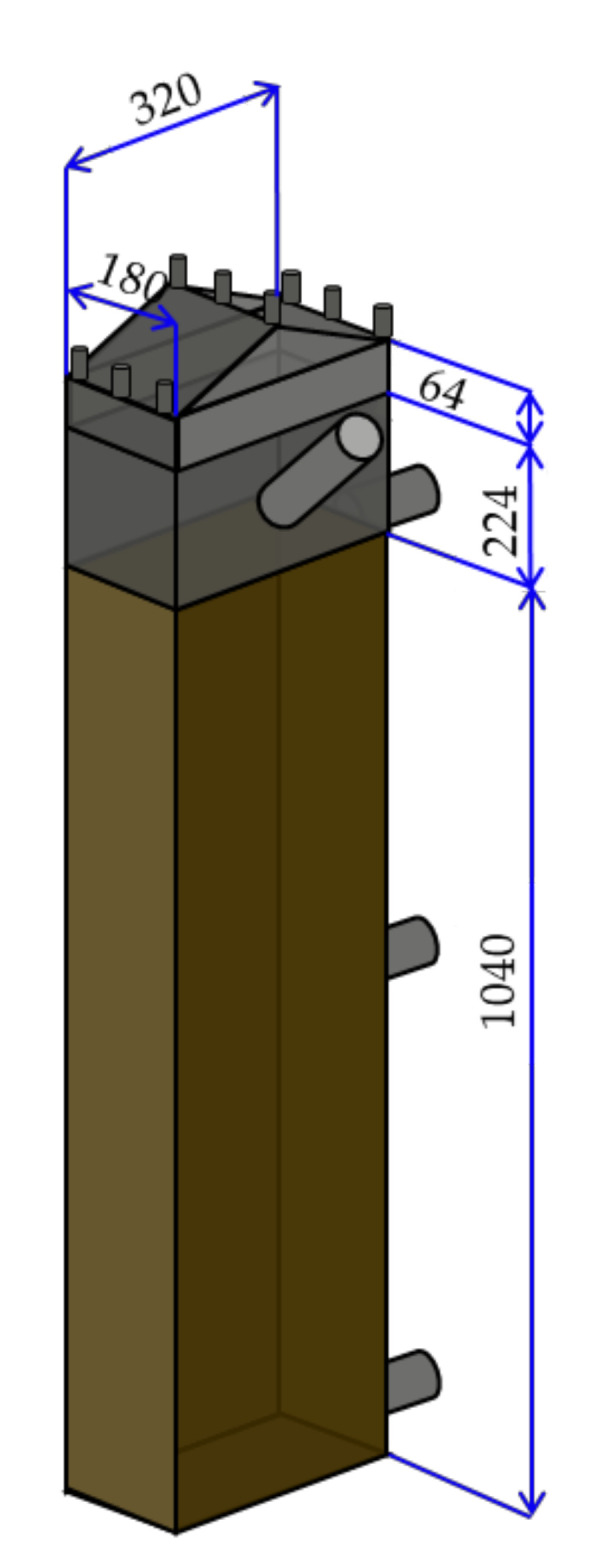
Specifications of the lab-scale swine barn.

Although the swine barns were designed considering the lab-scale study, the depth of the slurry pits reflected actual farm conditions (Figure 6). The working volume of the slurry pit swine barn was 60 L (length 320 mm, width 180 mm, and depth 1040 mm). The CT slurry pit was equipped with three ports; the upper port was used to circulate bio-liquor from the bioreactor to the slurry pit, and the lower port was used to transport the swine manure to the bioreactor as well as for sampling, while the middle one was used solely for sampling. On the other hand, the NCT slurry pit had two sampling ports located at the middle and the bottom of the pit. The height above the slurry surface in the slurry pit (224 mm) was used as the spare volume, and the swine manure was injected using a port located at the intermediate point of the spare volume.

#### 2.1.4. Collection and Input of Raw Swine Manure

The raw swine manure used in this study was collected from a swine farm located at Gapyeong-gun, Gyeonggi-do, Korea. The characteristics of swine manure are demonstrated in Table 1. The swine manure of the growing pigs is known for its high pollutant load and high level of odor emission due to high dietary crude protein concentrations [46,47,48]. Therefore, swine manure of the growing pigs was used in this study. The amount of swine manure input (0.43 L/d) in the slurry pits was calculated based on the actual farm conditions [39].

#### 2.1.5. Acid Absorption of Ammonia Gas

**Figure 7:**
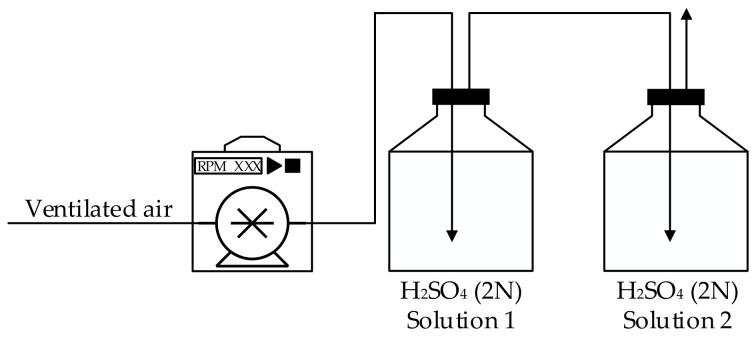
Schematic of gas capture process in lab-scale simulation.

In this study, NH_3_ was measured to elucidate the performance of real-time controlled BCS on reducing odor emission. The air ventilated in the bran was bubbled into the gas-trapping solution (2N sulfuric acid, H_2_SO_4_) to measure NH_3_ emission. The schematic of the gas-trapping process is presented in Figure 7. The gas trap solution was replaced with a new solution every week. On the opposite side of the ventilation line, an airflow line was installed to maintain fresh air flow into the lab-scale swine barn.

#### 2.1.6. Operation of the Lab-Scale Simulation System

Prior to the onset of the simulation experiment, the bioreactor and CT slurry pit were filled with the same mixed bio-liquor. The characteristics of bio-liquor are shown in Table 2. The mixed bio-liquor was collected from an aerobic tank of the activated sludge process of a swine farm located at Chuncheon-si, Gangwon-do, Korea.

In the BCS, the bioreactor plays a prominent role in improving the properties of the swine manure in the slurry pit. Therefore, the operating conditions for the experiment were set based on the bioreactor and the entire system was operated accordingly. The operating conditions applied to the lab-scale study are presented in Table 3. A volume of 1.2 L (2% and 4% of the slurry pit and bioreactor volume, respectively) of swine manure was circulated between the CT slurry pit and bioreactor in every cycle by the control algorithm. The circulation amount from the bioreactor to the slurry pit was limited to 0.77 L/cycle once a day to prevent overflow of bio-liquor due to raw swine manure input. During that time, as about 0.43 L of raw swine manure was injected into the slurry pit, the same volume of bio-liquor (0.43 L) was removed from the CT slurry pit first and stored in a storage tank to maintain the balance of the system. On the other hand, conventional swine farms, in reality, do not inject bio-liquor into the slurry pit. Therefore, to reflect the actual swine farm practice, in the NCT slurry pit only about 0.43 L of raw swine manure was injected once a day without bio-liquor.

### 2.2. Sampling Procedure 

In the lab-scale simulation, sampling was performed weekly using the sampling ports in the slurry pits. In the CT slurry pit, a total of 400 mL manure was collected using sampling ports. After vigorous mixing, a 200 mL sample was kept in the refrigerator at below 4 °C for further analysis and the rest was put back into the slurry pit through the manure input port. The NCT slurry pit was completely emptied at the beginning of the experiment and the sampling was conducted using the bottom port only. Therefore, 400 mL of manure was collected through the bottom port and homogenized. Of the total collected sample, 200 mL was used for further analysis and the rest was transferred back into the slurry pit. When the slurry pit was filled sufficiently, sampling was conducted as similar to CT. Bio-liquor samples were collected directly from the bioreactor inlet. During sampling, the time-profiles of ORP and pH (mV) in the bioreactor were carefully observed to predict the point of circulation. After finishing the circulation, 200 mL of the bio-liquor was sampled. 

### 2.3. Analytical Method

The preserved samples (200 mL) were mixed adequately and then divided into 100 mL each of the original and filtered liquid samples. The original samples were used to analyze the total solids (TS), total volatile solids (TVS), total suspended solids (TSS), total volatile suspended solids (TVSS) and total nitrogen (T-N). TS was measured after drying for 24 h at 105 °C, and then TVS was measured after burning at 550 °C for 4 h using a muffle furnace. For analyzing TSS, the samples were filtered using a glass fiber filter. The materials remaining on the filter paper were dried at 105 °C for 24 h to measure their weight. After that, the dried glass fiber filter was burned completely for 4 h at a temperature of 550 °C, and the weight of the remaining materials was measured to determine the TVSS. To analyze the TKN, the samples were digested with sulfuric acid at 380 °C for at least 4 h and then diluted into an appropriate concentration range with distilled water. After that, TKN was analyzed using an auto-analyzer (QuikChem 8500, Lachat, USA). 

The filtered liquid samples were used for STOC (soluble total organic carbon), NH_4_-N, NO_x_-N and T-N analysis. The STOC analysis was conducted using an automated TOC analyzer (Torch, Teledyne Tekmar, USA). The NH_4_-N, NO_x_-N, and T-N were measured using autoanalyzer (QuikChem 8500, Lachat, USA) after dilution. All the manure property analyses were performed following the standard methods [49]. To quantify NH_3_ emission from the lab-scale slurry pit, the TKN of H_2_SO_4_ solution was analyzed using an autoanalyzer (QuikChem 8500, Lachat, USA). 

## 3. Results and Discussion

### 3.1. Anoxic and Aerobic Phase Control Using ORP and pH (mV) Time-Profiles 

**Figure 8:**
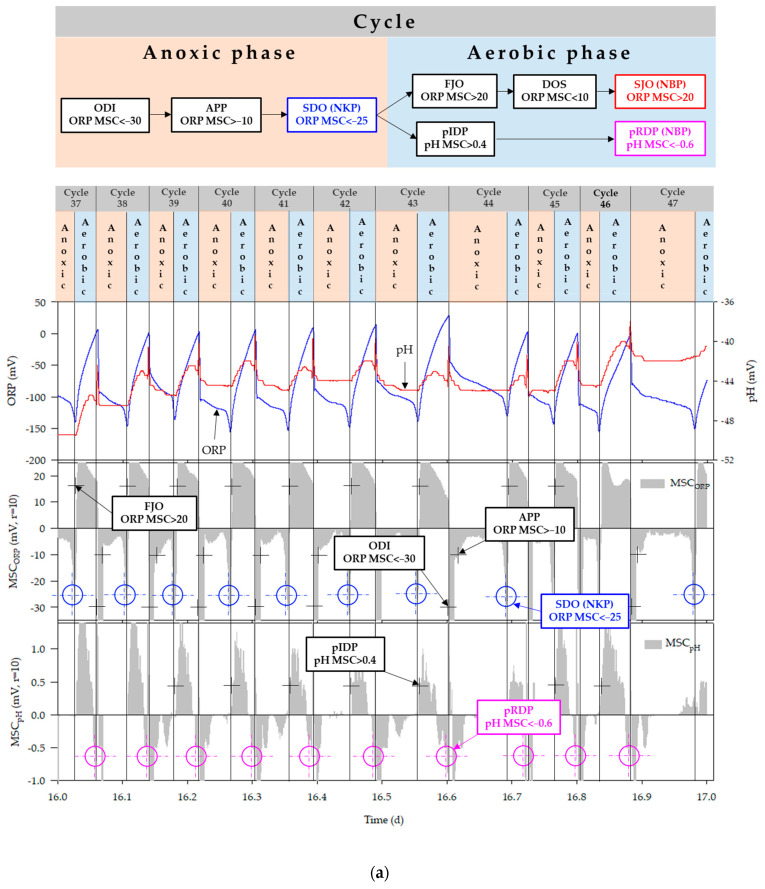

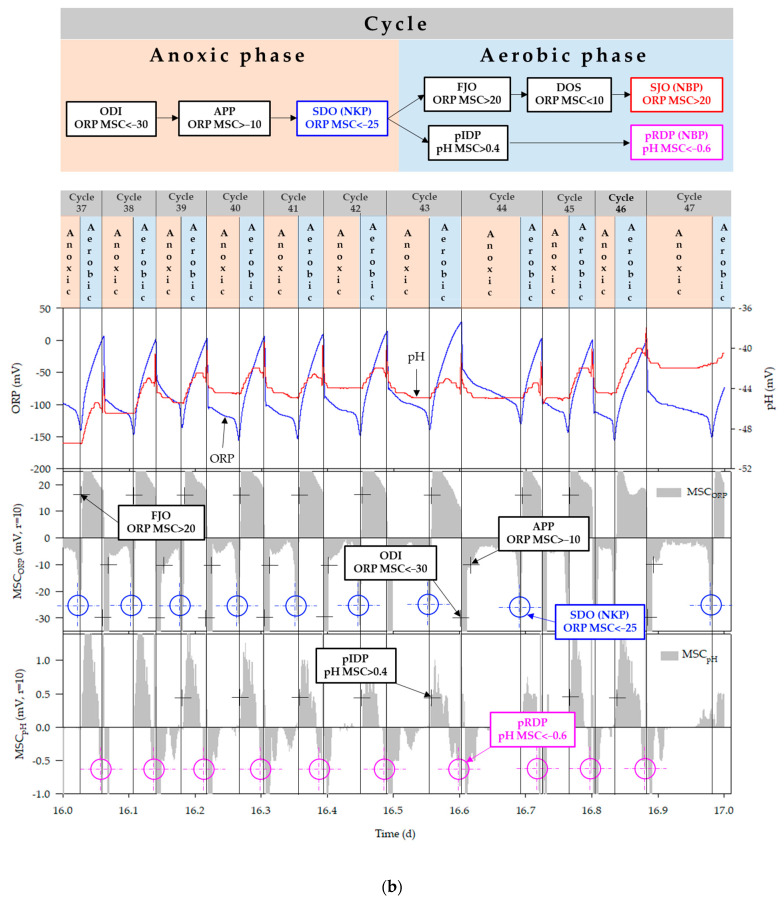

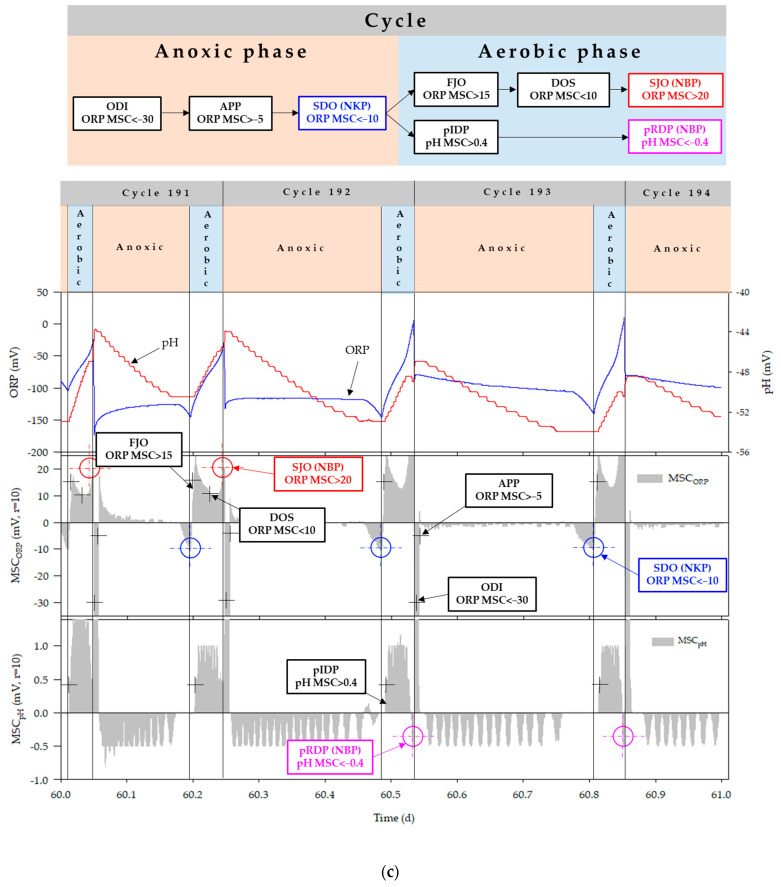

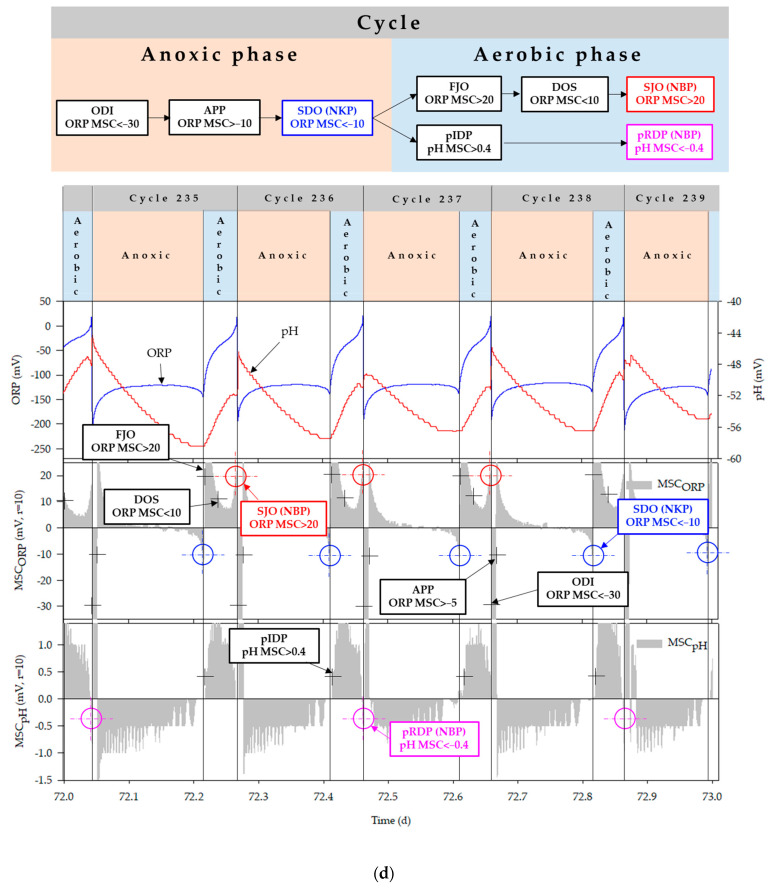
ORP and pH (mV) time-profiles in lab-scale bioreactor (**a**) day 16–17; (**b**) day 27–28; (**c**) day 60–61; (**d**) day 72–73.

**Figure 9:**
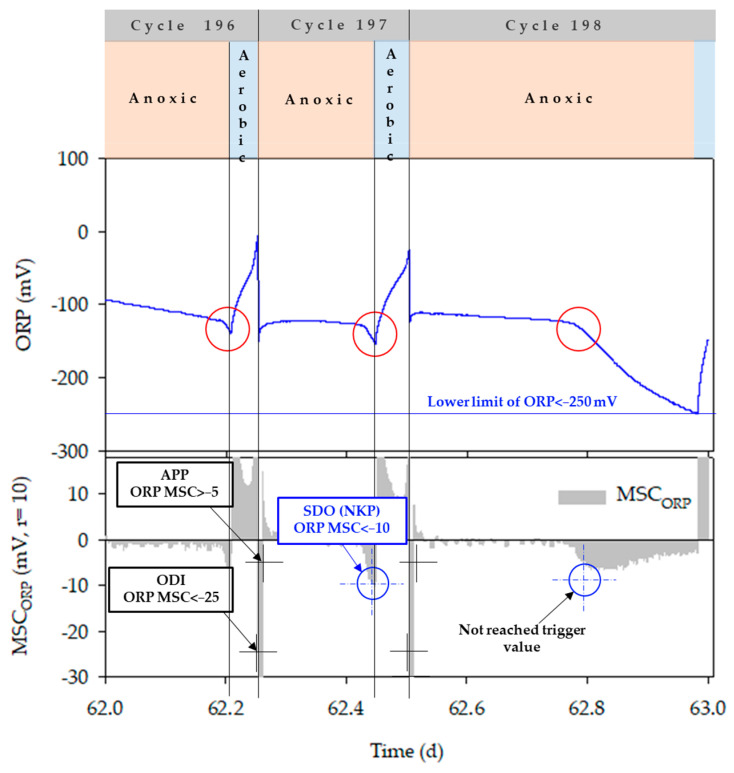
Anoxic phase control using ORP lower limit.

The performance of the ORP and pH (mV) time-profiles in controlling anoxic and aerobic conditions during the bioreactor operation is shown in Figure 8a–d. All the combinations of FDO, APP, and SDO trigger values were effective in controlling the anoxic phase in the bioreactor, regardless of ORP values, and ultimately resulted in successful detection of NKP during bioreactor operation using the ORP time-profile. This finding is in agreement with earlier studies [42,43,50]. Zanetti et al. reported that ORP values in the anoxic and the anaerobic conditions showed marked difference and therefore could be used to control the anoxic phase [51]. However, if the NKP cannot be detected through slope change for some reason, a control method using an absolute value may also be used. Kim found that ORP values varied from −200 to −100 mV in the presence of NO_x_-N and dropped rapidly to around −300 mV after completing the denitrification process in the bioreactor [39]. According to Kishida et al., the ORP dropped sharply below −300 mV in anaerobic conditions after being maintained around −100 to −200 mV in anoxic conditions [52]. Another study mentioned that when NO_x_-N was present in the system, the ORP stayed around −50 to −150 mV and decreased below −300 mV with the start of the anaerobic condition [53]. Won and Ra observed that the ORP in the anoxic condition ranged from 150 to −20 mV, but after initiation of the anaerobic condition, the ORP deteriorated to below –280 mV [43]. In cycle 198, the anoxic phase was controlled by an absolute ORP value (<−240 mV) (Figure 9). In that case, MSC_ORP_an_ decreased to the NKP point but did not reach the SDO trigger value. The ORP value slowly decreased until –250 mV, which was the lower limit for anoxic phase control. Although the time was delayed by approximately 4.8 h, the complete stop of the bioreactor operation was prevented by using the ORP lower limit. During the latter part of the experiment, the reduction in ORP by manure inflow dropped to nearly −220 mV until reaching the plateau point (Figure 8d). Therefore, to prevent the abnormal condition in the manure inflow, tracing of the ORP lower limit should be performed after 15 to 20 min from the circulation.

In the aerobic phase, time-profiles of ORP and pH (mV) separately and combinedly were used to detect the NBP. The example of single NBP detection by pH (mV) time-profile is shown in Figure 8a. In Figure 8a, the NBP was detected based on the pH (mV) time-profile, while only the FJO was identified on the ORP time-profile. Another case was observed in Figure 8c. In the case of cycles 192 and 193, because the decrease in the ORP slope was not enough, the MSC_ORP_ae_ did not fulfill the conditions required for the DOS trigger value. Hence, the algorithm could not detect NBP, although MSC_ORP_ae_ showed that SJO and the NBP was sensed on pH (mV) time-profile. Moreover, other examples of NBP detection by pH (mV) time-profile only were found in the cycles of 100, 101 (Figure 8b) and 238 (Figure 8d). In these cases, both FJO and DOS were detected by MSC_ORP_ae_ but the SJO trigger value was not reached and the NBP was detected by MSC_pH_ae_. On the other hand, the examples of NBP detected by MSC_ORP_ae_ were evident in Figure 8c,d. In the cycles of 190 and 192 (Figure 8c), and 235 and 237 (Figure 8d), the NBP were spotted on the ORP time-profile and the bioreactor was governed by the MSC_ORP_ae_ monitoring algorithm. Simultaneous appearances of the NBP on the ORP and pH (mV) time-profiles rarely found, and such a case was observed in cycle 236 in Figure 8d.

The results revealed that ORP was an effective control factor for the anoxic phase, but it was considered to be somewhat less stable to be used as a controlling factor for the aerobic phase. Unlike the MSC_pH_ae_, MSC_ORP_ae_ always shifted towards the positive region and resulted in unclear slope change, which was difficult to detect. In the case of the MSC_ORP_ae_, the decrease in slope was not enough after FJO, and therefore, the MSC_ORP_ae_ monitoring algorithm could not recognize the SJO. Such a situation can be a big problem for bioreactor control (Figure 8a). This problem is related to the variation in loading rate to the bioreactor [42]. Table 4 shows the influent loading rates corresponding to Figure 8a–d. In the case of 16~17 and 27~28 days, when aerobic conditions were controlled by pH (mV) time-profile, most of the loading rates were lower than 60~61 and 72~73 days. When the NH_4_-N loading was extremely low, the complete oxidation of NH_4_-N finished very quickly. Hence, the SJO could not be detected after FJO, and NBP disappeared on the ORP time-profile. Due to lack of appropriate operating strategies, the operational efficiency of BCS was often reduced. However, none of the studies have applied a real-time control strategy for the BCS operation. The importance of the results of this study thus showed the potency of real-time control process based on ORP and pH (mV) time profiles as operational parameters for efficient BCS operation in swine farms. 

### 3.2. Performance Evaluation of the Bioreactor 

Table 5 elucidates the quantitative results of the bioreactor operation. During the experiment, on average 3.9 ± 2.7 cycles/d were operated using the real-time control system. The circulation rate in the bioreactor depended on the completion of the denitrification process. In general, during the anoxic phase, swine manure is used as a carbon source because of its high organic matter content, and therefore, a short time is required to complete the denitrification process. However, in this study, the average duration of anoxic condition in the bioreactor was 19.4 ± 2.9 h/d. The long-term anoxic condition indicated that there was a delay in the completion of denitrification due to the shortage of useful carbon sources in circulated swine manure. The organic material in the slurry pit was assumed to be stable because of the continuous circulation of bio-liquor, and ultimately inhibited the denitrification reaction in the anoxic condition. The organic matter in swine manure was oxidized by aerobic bacteria and converted to a more stable organic material. The stable organic material was not suitable to be used as a carbon source for denitrification. Therefore, in the case of wastewater treatment processes, methanol as a carbon source is added for facilitating denitrification after aeration.

In this study, qualitative aspects of bioreactor were evaluated based on NH_4_-N, NO_X_-N and STOC. Table 6 shows the NH_4_-N removal performance of the bioreactor. Although the NH_4_-N concentration in the inflowed swine manure varied from 158.6 to 344.8 mg/L depending on the loading rate, no NH_4_-N was detected in the bio-liquor. The average NO_X_-N concentration in the circulated bioreactor was 8.3 mg/L; therefore, it can be said that most of the NH_4_-N (about 90%) from swine manure was oxidized to odorless NO_X_-N with little NH_3_ emission from the bioreactor. In addition, some of the NH_3_ in the bio-liquor can be also used for the cellular synthesis of microorganisms [54]. Thus, the direct emission of NH_3_ from the bioreactor might be very low. 

On the other hand, the STOC concentration in circulated bio-liquor was represented as 74.8% of inflowed swine manure (Table 6). Unlike NH_4_-N, the removal efficiency of STOC (organic matter) showed lower removal efficiency. In the aerobic process, the aerobic bacteria oxidized organic materials to carbon dioxide (CO_2_), but some other organic carbon-based compounds, such as humic and fulvic acid, proteins, nucleic acid, enzymes, structural components of cells, and products of energy metabolism can be generated [55,56]; hence, a reduction in removal efficiency can be observed. 

Studies also reported the necessity to apply advanced oxidation processes for the removal of color-causing materials and humic-type organic substances generated in the activated sludge process because they are non-biodegradable [57,58]. This is also thought to be related to the inhibition of the denitrification reaction by non-proper carbon sources as described above. Therefore, stable forms of organic matter (non-biodegradable organics) produced during bio-reaction can have a positive effect on the reduction in odor in the slurry pit.

### 3.3. Improvement of Swine Manure Properties in the Slurry Pit

In this study, soluble matters such as NH_4_-N and STOC were mainly considered to elucidate the improvement of swine manure properties in the slurry pit. Although other parameters such as TS, TVS, TSS, TVSS and T-N were also analyzed, it was not possible to perform an exact comparison due to difficulties in collecting representative samples from the entire slurry pit. 

Even though 0.43 L of raw swine manure was loaded into the slurry pit every day, the NH_4_-N concentration in the CT slurry pit was very low. The average removal efficiency of NH_4_-N in the CT slurry pit was found to be 92.8% (Table 7). However, an increase in the NH_4_-N concentration was observed in the NCT slurry pit. It is worth mentioning that the concentrations of NH_4_-N in raw swine manure loaded into the slurry pit were different during the study. 

Similar to NH_4_-N, the concentrations of STOC were found to be low in the CT slurry pit with an average removal efficiency of 78.4% (Table 7). Organic matter in the slurry pit is biodegraded to low-molecular organic compounds by anaerobic bacteria during long-term residence [15] and results in odor emission. Proper management strategies are therefore required to minimize odor emission due to longer retention times. Decreasing organic substances in the slurry pit through the circulation of the stabilized bio-liquor under the aerobic condition and alleviating the anaerobic condition can be useful in reducing harmful gases inside the swine barn. 

In general, the manure properties of the CT slurry pit showed remarkable improvement (more than 85% except for STOC and TS) compared to the NCT slurry pit. Such a phenomenon was observed due to the bioreactor/slurry pit size ratio. However, the real-time control system using the time-profiles of ORP and pH (mV) always ensured the optimized operating conditions regardless of the bioreactor/slurry pit size ratio.

### 3.4. NH_3_ Reduction Efficiency

Intensive swine farming practices extensively contribute to NH_3_ emission [59,60]. NH_3_ emitted from swine farms decreases the swine productivity and affects the people working in the farms and living in neighborhoods nearby [61]. Table 8 elucidates the variations in NH_3_ emission from CT and NCT slurry pits. As a result of analyzing the gas trap solution, a high level of NH_3_ reduction efficiency (around 98.3%) was observed in the CT slurry pit.

**Figure 10:**
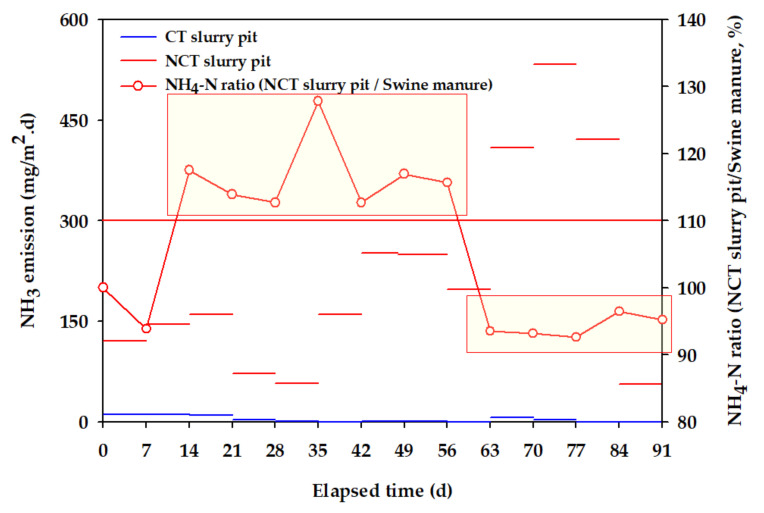
NH_3_ emission trend of CT and NCT slurry pits in the lab-scale simulation.

Figure 10 demonstrates the NH_3_ emission trend of CT and NCT slurry pits during this study. NH_3_ emission from the CT was much lower than the NCT. In the NCT slurry pit, around 12% higher NH_4_-N concentration was found compared to raw swine manure during 14–56 days (Figure 10). Decomposition of organic nitrogen in swine manure in the NCT slurry pit might result in high NH_4_-N concentration. However, a decrease in NH_4_-N concentration was also observed during 63–91 days. At that time, NH_4_-N in the slurry pit converted to NH_3_ gas and it coincided with the time when the NH_3_ emission increased from the NCT slurry pit.

Track studies were also conducted every 24 h to observe the trend of NH_3_ emission from the slurry pits in more detail (data not shown). The NH_3_ emissions from the slurry pit at any time can fluctuate due to various and complex factors such as swine diets, precursor concentration, temperature, pH, surface air flow, and scum layer formation and thickness [61,62,63,64]. These characteristics made it difficult to manage the air quality in the swine barn through accurate prediction. The results showed that NH_3_ emission between 56–63 days was 0.4 and 172.8 mg/m^2^·d from CT and NCT slurry pits, respectively (Figure 10). In contrast, the NH_3_ emission trend between 77–91 days showed a slightly different pattern from days 56–63. NH_3_ emission from the NCT slurry pit in days 77–84 remained constant (378.7 mg/m^2^·d), whereas from day 84, it suddenly decreased to 53.7 mg/m^2^·d. To sum up, it can be said that NH_3_ emission was effectively reduced by improving the swine manure properties in the real-time controlled CT slurry pit.

## 4. Conclusions

The real-time control technology using moving slope changes of ORP and pH(mV) time profiles was effective in controlling the anoxic and aerobic phases during the bioreactor operation. The ORP time profile successfully detected the NKP in the anoxic phase and was less stable in detecting NBP in the aerobic phase. On the other hand, the pH time profile showed a more efficient detection of NBP. The performance evaluation of the bioreactor revealed that because most of the NH_4_-N (around 90%) from swine manure was oxidized to odorless NO_X_-N, the direct emission of NH_3_ from the bioreactor might be very low. Production of organic substances during the aerobic phase resulted in a reduced STOC removal efficiency. Regarding manure properties improvement in the slurry pit, removal efficiencies of NH_4_-N and STOC and other analyzed parameters were much higher in the CT slurry pit than the NCT slurry pit. In addition, about 98.3% of NH_3_ can be removed from the CT slurry pit through manure properties improvement. The results of this lab-scale study therefore indicate the efficacious application real-time control strategy in operating BCS and encourage further studies. 

## Figures and Tables

**Table 1:** Characteristics of the swine manure used in this study.

Parameter	Concentration (mg/L)
TS ^1^	46,179.2 ± 22,581.9
TVS ^2^	33,227.1 ± 17,742.2
TSS ^3^	39,567.5 ± 24,987.0
TVSS ^4^	30,565.0 ± 19,152.8
STOC ^5^	2719.3 ± 522.8
NH_4_-N ^6^	12,305.8 ± 5040.0
T-N ^7^	5187.4 ± 2015.2

^1^ TS, total solid; ^2^ TVS, total volatile solid; ^3^ TSS, total suspended solid; ^4^ TVSS, total volatile suspended solids; ^5^ STOC, soluble total organic carbon; ^6^ NH_4_-N, ammonium nitrogen; ^7^ T-N, total nitrogen.

**Table 2:** Characteristics of the bio-liquor used in this study.

Parameters	Concentration (mg/L)
TS ^1^	12,616.7 ± 23.6
TVS ^2^	6200.0 ± 47.1
TSS ^3^	7244.4 ± 126.2
TVSS ^4^	5444.4 ± 167.8
STOC ^5^	1132.0 ± 43.0
NH_4_-N ^6^	20.1 ± 1.5
NO_X_-N ^7^	ND ^10^
TKN ^8^	1230.3 ± 5.6
T-N ^9^	1569.1 ± 12.7

^1^ TS, total solid; ^2^ TVS, total volatile solid; ^3^ TSS, total suspended solid; ^4^ TVSS, total volatile suspended solids; ^5^ STOC, soluble total organic carbon; ^6^ NH_4_-N, ammonium nitrogen; ^7^ NO_x_, Nitrogen oxides; ^8^ TKN, total kjeldahl nitrogen; ^9^ T-N, total nitrogen; ^10^ ND, Not detected.

**Table 3:** Operational conditions of the bioreactor and slurry pits in the lab-scale simulation system.

Parameters	Bioreactor	CT Slurry Pit	NCT Slurry Pit
Working volume (L)	30	60	60
Initial condition	filled with bio-liquor	filled with bio-liquor	empty
Manure input (L/d)	-	0.43	0.43
Circulation rate (L/cycle)	1.2	1.2	-
Circulation rate based on volume (%/cycle)	4	2	-
Aeration rate (L/L·min)	0.05	-	-

**Table 4:** Loading rate in the lab-scale bioreactor.

Parameter	Bioreactor Loading Rate (kg/m^3^/Cycle)						
	TS ^1^	TVS ^2^	TSS ^3^	TVSS ^4^	STOC ^5^	NH_4_-N ^6^	T-N ^7^
16~17 (d)	0.393~0.418	0.153~0.158	0.163~0.171	0.107~0.125	0.072~0.073	0.005~0.006	0.011~0.014
27~28 (d)	0.518~0.630	0.169~0.184	0.132~0.151	0.106~0.122	0.075~0.077	0.007~0.009	0.016~0.018
60~61 (d)	0.471~0.484	0.163~0.191	0.253~0.261	0.154~0.186	0.079~0.081	0.011~0.013	0.021~0.024
72~73 (d)	0.465~0.471	0.155~0.172	0.214~0.248	0.147~0.149	0.091~0.102	0.009~0.011	0.028~0.024

^1^ TS, total solid; ^2^ TVS, total volatile solid; ^3^ TSS, total suspended solid; ^4^ TVSS, total volatile suspended solids; ^5^ STOC, soluble total organic carbon; ^6^ NH_4_-N, ammonium nitrogen; ^7^ T-N, total nitrogen.

**Table 5:** Quantitative information of the bioreactor operation.

Parameter		Value
Duration	anoxic phase (h/d)	19.4 ± 2.9
	aerobic phase (h/d)	4.6 ± 2.9
Number of circulation	total (cycle)	311
	average (cycle/d)	3.9 ± 2.7
Circulation rate	based on bio-reactor volume (%/d)	15.7 ± 10.9
	based on slurry pit volume (%/d)	7.9 ± 5.5

**Table 6:** NH_4_-N and STOC removal performance of the bioreactor in lab-scale simulation.

Parameter	Swine Manure (mg/L)				Bio-Liquor (mg/L)						Bio-Liquor /Swine Manure (%)
	Average	Min.	Max.	Std. Dev.	Average	Min.		Max.	Std. Dev.		
NH_4_-N ^1^	229.7	158.6	344.8	61.2	ND ^4^					-	
NO_X_-N ^2^	ND ^4^				8.3	2.3	15.7		2.3		-
STOC ^3^	2100.0	1795.7	2694.4	335.4	1570.9	1071.8		1968.0	312.0		74.8

^1^ NH_4_-N, ammonium nitrogen; ^2^ NO_x_, Nitrogen oxides; ^3^ STOC, soluble total organic carbon; ^4^ ND, not detected.

**Table 7:** Swine manure properties in the CT and NCT slurry pits.

Parameter	CT Slurry Pit	NCT Slurry Pit	Reduction Efficiency (%)
TS ^1^	9233.3 ± 1663.9	71,650.9 ± 39,616.6	87.1
TVS ^2^	2585.8 ± 1219.9	50,362.1 ± 28,798.2	94.9
TSS ^3^	2817.6 ± 1579.0	64,494.9 ± 39,647.0	95.6
TVSS ^4^	1961.5 ± 1174.6	47,082.9 ± 28,420.9	95.8
STOC ^5^	2047.1 ± 372.2	9461.3 ± 5350.3	78.4
NH_4_-N ^6^	208.8 ± 81.9	2893.2 ± 612.4	92.8
T-N ^7^	472.5 ± 233.5	5350.0 ± 1691.4	91.2

^1^ TS, total solid; ^2^ TVS, total volatile solid; ^3^ TSS, total suspended solid; ^4^ TVSS, total volatile suspended solids; ^5^ STOC, soluble total organic carbon; ^6^ NH_4_-N, ammonium nitrogen; ^7^ T-N, total nitrogen.

**Table 8:** NH_3_ emission from the CT and NCT slurry pits in the lab-scale simulation system.

Parameter	CT Slurry Pit	NCT Slurry Pit	Reduction Efficiency (%)
Total emission (mg/m^2^)	340.4	19,850.0	98.3
Average emission (mg/m^2^·d)	3.7 ±4.4	218.1 ±151.2	
Maximum emission (mg/m^2^·d)	11.6	245.2	
Minimum emission (mg/m^2^·d)	0.03	54.2	

## Supplementary Materials

The following are available online at https://www.mdpi.com/2076-2615/11/2/311/s1, Table S1: Calculation method of ventilation amount in lab-scale swine barn [45].
