# Supplementary material for: Development of a Real-Time Controlled Bio-Liquor Circulation System for Swine Farms: A Lab-Scale Study
Source: Animals (Basel). 2021 Jan 26;11(2):311. doi: 10.3390/ani11020311 (PMC7910917; doi:10.3390/ani11020311)
Supplement: Supplementary file 1 [file animals-11-00311-s001.pdf]

Supplementary Table 1. Calculation method of ventilation amount in lab-scale swine barn [45]

**Parameters**		**Breeding step**	**Spring and fall**	**Winter**	**Summer**
**Ventilation rate (m^3/h)**	Standard swine barn	Sow	150 x head	Spring and fall x 15%	Spring and fall x 350%
		Piglet	1.3 x head x BW[1]		
		Growing	60 x head		
		Fattening	110 x head		
	This study [2]	Growing	5.0	0.8	17.5
		Fattening	9.2	1.4	32.2
		Average	7.1	1.1	24.9
Ventilation rate (m^3/d) = Average ventilation rate (m^3/h) x Sidewall height ratio of lab-scale swine barn / standard swine barn* x 24 h/d			4.4	0.7	15.3

[1] BW, body weight; [2] No. of pig heads = 0.084; * sidewall height ratio of lab-scale swine barn / standard swine barn = 0.0256
